# 免疫介导的再生障碍性贫血小鼠模型造模过程中病理性T细胞的特征分析

**DOI:** 10.3760/cma.j.issn.0253-2727.2022.07.010

**Published:** 2022-07

**Authors:** 惠 贾, 赠华 林, 玟 李, 志鹏 张, 红 刘

**Affiliations:** 南通大学医学院，南通大学附属医院，南通 226001 Medical School, Nantong University, Affiliated Hospital of Nantong University, Nantong 226001, China

**Keywords:** 贫血，再生障碍性, 小鼠模型, 病理性T细胞, 归巢, 特征, Anemia, aplastic, Mouse model, Pathogenic T cells, Homing, Characterization

## Abstract

**目的:**

通过DsRed小鼠淋巴细胞输注加全身辐照（TBI）建立免疫介导的再生障碍性贫血（AA）C.B10-H2b/LilMcd (C.B10)小鼠模型，分析C.B10 AA小鼠模型中病理性T细胞在不同器官的动态归巢过程及特征。

**方法:**

通过异体淋巴细胞输注加TBI建立AA小鼠模型，以TBI组为对照，分析外周血细胞和骨髓单个核细胞数，验证造模效果。在造模的第3、6、9、12天，处死小鼠并收集骨髓、脾脏、淋巴结和胸腺单个核细胞，流式细胞术分析DsRed^+^ T细胞的动态变化及效应记忆T细胞（T_EM_）、中央记忆T细胞（T_CM_）、初始T细胞（naïve T）和效应T细胞（T_EFF_）的比例。PCR Array分析AA小鼠不同组织内DsRed^+^病理性T细胞活化相关基因的表达谱。

**结果:**

与TBI组相比，AA组小鼠在造模第9、12天出现严重骨髓衰竭，第12天时骨髓有核细胞数、外周血细胞数均显著减少（*P*值均<0.05）。AA组小鼠骨髓、脾脏、淋巴结内DsRed^+^ T细胞比例随时间增加而增加。骨髓中的DsRed^+^ T细胞比例在第3、6天低于脾脏与淋巴结，但在第12天时高于脾脏与淋巴结（*P*值均<0.05）。整个骨髓衰竭形成过程中，胸腺中DsRed^+^ T细胞比例均最低。在第12天时，AA小鼠骨髓、淋巴结和脾脏单个核细胞中，DsRed^+^CD3^+^CD4^+^ T细胞比例分别为（91.38±2.10）％、（39.78±6.98）％、（67.87±12.77）％，三者之间两两比较均有统计学意义（*P*值均<0.05）；DsRed^+^CD3^+^CD8^+^ T细胞比例分别为（98.21±1.49）％、（94.06±4.20）％、（96.29±1.23）％，差异均无统计学意义（*P*值均>0.05）。第9、12天，AA组小鼠骨髓中几乎所有的DsRed^+^CD4^+^ T或DsRed^+^CD8^+^ T细胞均为T_EM_，而淋巴结中包括T_EM_、T_CM_以及naïve T。PCR Array结果显示：骨髓中DsRed^+^CD4^+^或DsRed^+^CD8^+^ T细胞中CD38、IFN-γ、LAG3、CSF1、SPP1及TNFSF13B表达增高，但 DsRed^+^CD4^+^ T细胞中FOXP3、CTLA4表达降低。

**结论:**

DsRed小鼠淋巴细胞输注可导致C.B10小鼠发生骨髓衰竭，并可以在该AA模型小鼠中追踪DsRed^+^病理性T细胞。在C.B10 AA模型小鼠体内，DsRed^+^CD8^+^和DsRed^+^CD4^+^ T细胞先归巢到脾脏与淋巴结，在其中扩增分化，最终转运至骨髓并转化为T_EM_。胸腺对异体T细胞的归巢过程更具抵抗性，提示胸腺在免疫介导AA模型的骨髓衰竭过程中可能是一个免疫豁免部位。在C.B10 AA模型小鼠的骨髓衰竭形成过程中，归巢到骨髓的DsRed^+^病理性T细胞较迁移到脾脏与淋巴结的细胞具有更强的免疫活性，而免疫抑制活性则减弱。

## Abstract (en)

**Objective:**

This study aims, in addition to characterizing pathogenic T cells trafficking to bone marrow (BM) and other organs, to establish immune-mediated AA C.B10 mouse model by DsRed mouse (B6 background) lymph nodes (LN) cells infusion after a total body irradiation (TBI).

**Methods:**

The C.B10 mice received a 5 Gy TBI and then were infused with DsRed mouse (B6 background) LN cells at 5×10^6^/mouse via a tail vein injection. The severity of bone marrow failure (BMF) was observed by mononuclear cell count in bone marrow and peripheral blood cell count. On days 3, 6, 9, and 12, mice were sacrificed and collected BM, spleens, LN, or thymus to analyze the dynamic change and activation status of donor T cells in these organs by a flow cytometry. At day 12, the donor-derived T cells from BM, spleens, and LN were sorted to collect the DsRed^+^CD3^+^CD4^+^ T cells and DsRed^+^CD3^+^CD8^+^ T cells for RNA isolation and gene expression analyses by PCR array.

**Results:**

Relative to control animals that received 5 Gy TBI without LN cell infusion, AA mice developed severe BMF with dramatic decrease in total BM cells, hemoglobin, white blood cells, and platelets in peripheral blood on days 9 and 12 after the LN cell infusion. The frequencies of DsRed^+^ T cells trafficking to BM, LN, and spleens increased with time. Surprisingly, although the DsRed^+^ T cells in BM increased dramatically at a level much higher than those in the spleens and LN on day 12, there were very few DsRed^+^ T cells in BM on days three and six, which was significantly lower than those in spleens or LN. The frequency of DsRed^+^ T cells in thymus was the lowest during the whole process. On day 12, the DsRed^+^CD3^+^CD4^+^ T cells of BM, LN, and spleens from AA mice were（91.38±2.10）％,（39.78±6.98）％, and（67.87±12.77）％, respectively. On the contrary, the DsRed^+^CD3^+^CD8^+^T cells of BM, LN, and spleens were（98.21±1.49）％,（94.06±4.20）％, and（96.29±1.23）％, respectively. We assessed the donor T cell phenotypes using the CD44 and CD62L markers and found that almost all of the DsRed^+^CD4^+^ or DsRed^+^CD8^+^ T cells in BM were effector memory T cell phenotype from day 9 to day 12. Meanwhile, transcriptome analyses showed higher expression in CD38, IFN-γ, LAG3, CSF1, SPP1, and TNFSF13B in BM DsRed^+^CD4^+^ and DsRed^+^CD8^+^ T cells. However, there was a lower expression in FOXP3 and CTLA4 in BM DsRed^+^CD4^+^ T cells than those in spleens and LN.

**Conclusion:**

The DsRed LN cells infusion to induce BMF in CB10 mice enabled to track the donor-derived pathogenic T cells. Besides previously published findings in this model, we demonstrated that donor CD4^+^ and CD8^+^ T cells primarily homed to spleens and LN, expanded and differentiated, then infiltrated in BM with a terminal effector memory phenotype. The T cells infiltrated in BM showed more activation and less immunosuppression characteristics compared to those homing to spleens and LN during the BMF development.

再生障碍性贫血（AA）是一种由多种原因引起的以骨髓造血衰竭和全血细胞减少为特征的骨髓造血衰竭（BMF）综合征[1]。AA的发病机制主要有造血干/祖细胞功能缺陷和数量异常[1]–[3]、造血微环境[4]–[5]及免疫因素异常[1],[6]–[7]。T淋巴细胞异常活化、功能亢进使造血细胞凋亡和造血功能衰竭是原发获得性AA的一个重要发病机制[8]–[9]。然而，异常活化的T细胞如何攻击造血干/祖细胞及在AA患者体内的分布情况尚有待进一步研究。

由于重型AA（SAA）患者的骨髓和外周血中的细胞数量极少，常难以取得足够供研究的样本，限制了对AA发病机制的深入研究。本研究基于既往理论和AA模型小鼠的制备经验[10]–[11]，选用供鼠淋巴细胞输注和TBI联用的方法，建立可以在不同器官中追踪供鼠病理性T细胞归巢过程的免疫介导的AA小鼠模型，了解病理性T细胞在AA发病过程中的动态变化，可能可以为AA发病机制的研究开辟一条新的途径。

## 材料与方法

1. 实验材料及仪器：IMDM培养基、RPMI 1640培养基、EDTA、ACK红细胞裂解液购自美国Gibco公司，PBS缓冲液购自美国Hyclone公司，各类流式抗体购自美国BD公司，RNA提取试剂盒、RNA纯化试剂盒、Activation RT^2^ Profiler PCR Array、RNeasy Kit、RNase free DNase Ⅰ、RT2 First Strand Kit、RT^2^ Profiler™ PCR Array Mouse Activation购自美国Qiagen公司，离心管、无菌试管等耗材购自美国Corning公司。FACS流式细胞仪购自美国BD公司，HemaVet 950动物血液分析仪购自美国Drew Scientific公司，Shepherd Mark1137铯γ源辐照设备购自美国J. L. Shepherd & Associates公司，384-孔型LightCycler 480实时PCR仪购自瑞士Roche公司。

2. 实验动物：实验用雌性C.B10小鼠（小鼠编号：001952，8～12周龄，体重18～22 g）、父代雄性和雌性DsRed转基因小鼠（小鼠编号：006051）均由美国国立卫生研究院心肺血液病研究所从美国Jackson实验室购买，实验用雌性DsRed小鼠为父代雄性和雌性DsRed转基因小鼠配种后的子一代雌性小鼠（8～12周龄，体重18～22 g）。采用随机数字表法将雌性DsRed小鼠随机分为TBI组（16只）和AA组（16只）。所有小鼠均在实验小鼠的标准生活条件下饲养。本实验所用动物实验均经过美国国立卫生研究院心肺血液病研究所动物实验管理委员会批准。

3. AA小鼠模型的建立：采集B6-Cg-Tg（CAG-DsRed*MST）1Nagy/J（DsRed）雌性小鼠（B6源）淋巴结制成单细胞悬液。C.B10雌性小鼠在5 Gy的全身辐照（TBI）4 h后通过尾静脉注射5×10^6^/只剂量的DsRed小鼠的淋巴结单个核细胞悬液造模。在注射异体淋巴细胞后第3、6、9、12 d采集模型小鼠眼眶静脉血和股骨骨髓单个核细胞，以单纯TBI处理的C.B10小鼠作为对照组。分析两组小鼠之间的外周血常规和骨髓单个核细胞计数，验证造模效果。

4. 供鼠来源病理性T淋巴细胞动态变化的监测：在输注淋巴细胞的第3、6、9和12天，处死AA模型小鼠并收集骨髓、脾脏、淋巴结和（或）胸腺单个核细胞，通过流式细胞术分析这些器官中供鼠来源病理性T细胞的动态变化，病理性T细胞主要包括：初始T细胞（naïve T）、效应记忆T细胞（T_EM_）、中央记忆T细胞（T_CM_）和效应T细胞（T_EFF_），免疫表型分别为CD44^−^CD62L^+^、CD44^+^CD62L^−^、CD44^+^CD62L^+^和CD44^−^CD62L^−^。

5. 供鼠来源病理性T淋巴细胞活化基因的表达：在输注淋巴细胞后的第12天，收集AA模型小鼠骨髓、脾脏和淋巴结的单个核细胞，通过流式细胞仪分选收集供鼠来源的CD3^+^CD4^+^ T淋巴细胞和CD3^+^CD8^+^ T淋巴细胞，分别以供鼠淋巴结CD3^+^CD4^+^和CD3^+^CD8^+^ T淋巴细胞为对照组，提取RNA，合成cDNA，进行实时荧光定量PCR反应，分析模型小鼠不同淋巴造血组织内供鼠来源的病理性T淋巴细胞活化相关基因的表达谱。

6. 统计学处理：采用GraphPad Prism 6.01软件进行绘图与统计分析。每组数据来自3次独立的实验，所有数据以均数±标准差表示，组间比较采用独立样本*t*检验或单因素方差分析，方差分析有意义的指标采用Tukey法进行组内多重比较。*P*<0.05为差异有统计学意义。

## 结果

一、DsRed小鼠淋巴结细胞诱导的C.B10小鼠AA模型的建立和鉴定

造模后第3、6天，AA组与TBI组外周血WBC、ANC、HGB、PLT及股骨单个核细胞计数差异均无统计学意义（*P*值均>0.05）；第9、12天AA组小鼠外周血WBC、ANC、PLT及股骨单个核细胞计数均明显低于TBI组（*P*值均<0.05）；第12天时AA组小鼠HGB也显著减少，与TBI组比较差异有统计学意义（*P*<0.05）；TBI组外周血各项指标第12天时接近TBI处理前水平，处于恢复状态，股骨单个核细胞计数在第3、6、9和12天差异均无统计学意义（图1）。

**图1:**
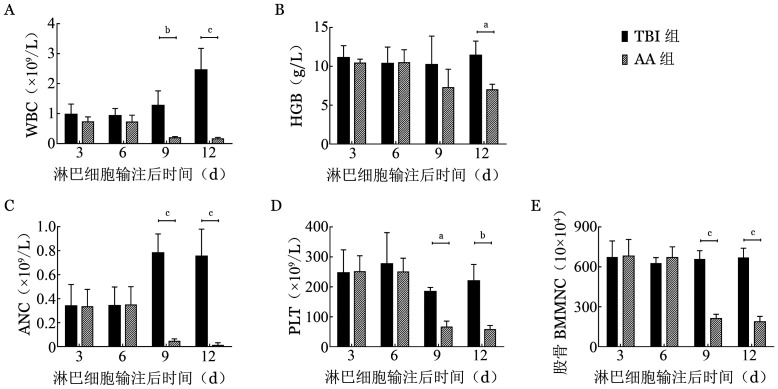
DsRed小鼠淋巴结细胞诱导的C.B10小鼠再生障碍性贫血模型不同时间外周血WBC（A）、HGB（B）、ANC（C）、PLT（D）与股BMMNC计数（E）结果（^a^*P*<0.05、^b^*P*<0.001、^c^*P*<0.0001） AA组：再生障碍性贫血模型小鼠；TBI组：全身辐照对照组；BMMNC：骨髓单个核细胞

二、病理性T细胞在不同器官的归巢过程分析

1. 供鼠来源T淋巴细胞在不同器官的分布：AA模型小鼠体内，骨髓、脾脏和淋巴结内DsRed^+^CD3^+^和DsRed^+^CD3^+^CD8^+^ T细胞在淋巴细胞群中比例随时间逐渐增加（图2）；第3、6天，骨髓和胸腺中的DsRed^+^CD3^+^ T细胞在淋巴细胞群内比例极低，在脾脏、淋巴结中的比例高于骨髓和胸腺；而在第12天时，DsRed^+^CD3^+^ T细胞在骨髓中的比例显著高于脾脏、淋巴结和胸腺，且在脾脏、淋巴结中比例也显著高于胸腺，差异均有统计学意义（*P*值均<0.05），整个骨髓衰竭形成过程中，胸腺内的DsRed^+^CD3^+^ T细胞在淋巴细胞群内的比例均最低（图2）。

**图2:**
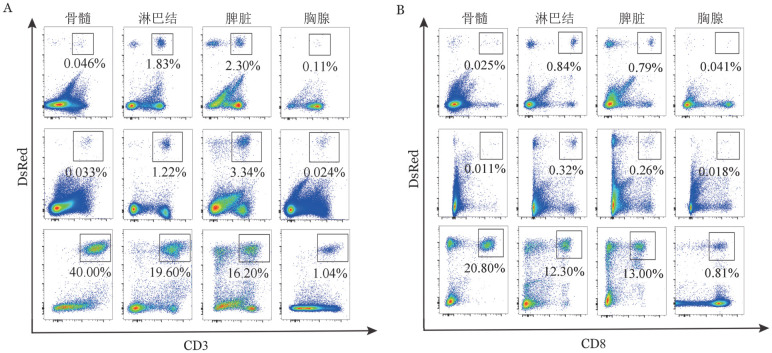
流式细胞术检测不同时间C.B10再生障碍性贫血模型小鼠体内DsRed^+^CD3^+^（A）和DsRed^+^CD3^+^CD8^+^（B）T细胞在不同器官的淋巴细胞内的表达

2. 供鼠来源CD3^+^CD8^+^H60^+^ T细胞毒性淋巴细胞在不同器官的分布：流式细胞检测结果分析显示：AA模型小鼠体内，骨髓、脾脏和淋巴结内特异性表达H60的CD8^+^细胞毒性T淋巴细胞（CD3^+^CD8^+^H60^+^）在T淋巴细胞中比例随时间增加而增加（图3）；第12天时，骨髓CD3^+^CD8^+^H60^+^ T细胞比例最高，而胸腺CD3^+^CD8^+^H60^+^ T细胞的比例在整个过程中均最低。

**图3:**
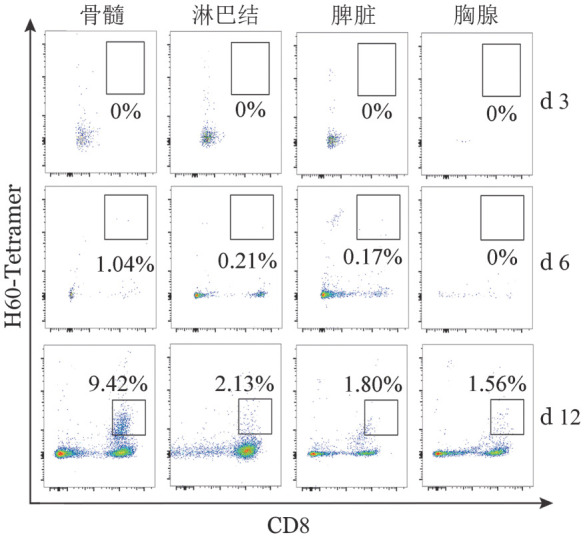
流式细胞术检测不同时间C.B10再生障碍性贫血模型小鼠体内特异性表达H60的CD8^+^细胞毒性T淋巴细胞在不同器官的表达

3. 供鼠来源CD4^+^、CD8^+^淋巴细胞在不同器官的比例分析：第12天时，AA模型小鼠骨髓、淋巴结和脾脏DsRed^+^CD3^+^CD4^+^ T细胞比例分别为（91.38±2.10）％、（39.78±6.98）％和（67.87±12.77）％，组间及组内两两比较均有统计学意义（*P*值均<0.05）；DsRed^+^CD3^+^CD8^+^ T细胞比例分别为（98.21±1.49）％、（94.06±4.20）％及（96.29±1.23）％，差异无统计学意义（*P*值均>0.05）。

4. 病理性T细胞在不同淋巴组织中的表型特征分析：第9、12天，AA模型小鼠骨髓中80％以上DsRed^+^ CD4^+^、DsRed^+^ CD8^+^ T细胞为T_EM_，淋巴结中的供鼠来源的CD4^+^ T细胞包括T_EFF_、T_EM_、T_CM_以及naïve T细胞（图4）。

**图4:**
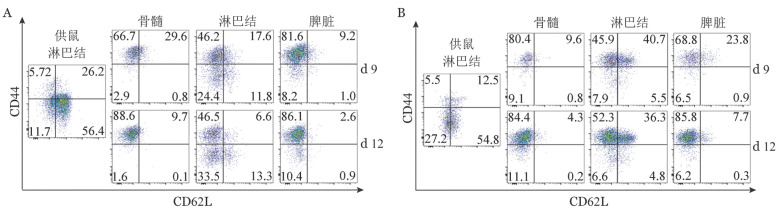
流式细胞术检测免疫介导的再生障碍性贫血小鼠中供鼠来源CD3^+^CD4^+^（A）和CD3^+^CD8^+^（B）T细胞CD44、CD62L表达

三、活化基因表达分析

第12天，AA模型小鼠中DsRed^+^CD4^+^T细胞在不同淋巴组织中的活化基因表达谱见图5，骨髓中DsRed^+^CD4^+^ T细胞及DsRed^+^CD8^+^T细胞中CD38、IFN-γ、LAG3、CSF1、SPP1及TNFSF13B基因相对表达水平上调；但和脾脏和淋巴结相比，骨髓中DsRed^+^CD4^+^ T细胞中FOXP3和CTLA4基因表达下调。

**图5:**
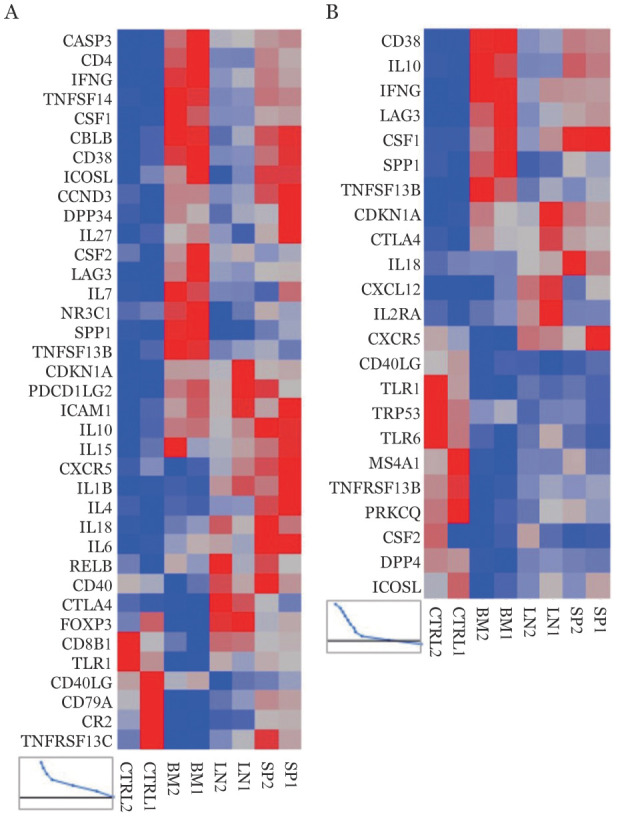
免疫介导的AA小鼠中供鼠来源CD4^+^（A）、CD8^+^（B）T细胞在不同淋巴组织中的活化基因表达 SP：脾脏；LN：淋巴结；BM：骨髓；CTRL：供鼠淋巴结

## 讨论

AA是一种以骨髓造血衰竭和全血细胞减少为特征的后天性异质性疾病，T淋巴细胞异常活化和功能亢进造成骨髓损伤在原发性获得性AA发病机制中占主要地位，但异常活化的T细胞如何攻击造血干细胞及在AA患者体内的分布情况尚有待进一步研究。

本研究建立可以在不同器官中追踪供鼠病理性T细胞归巢过程的免疫介导的AA小鼠模型，以便了解病理性T细胞在AA发病过程中的动态变化，并对其病理性T细胞的特征进行分析。实验结果显示该模型的发病时间和既往正常的B6小鼠淋巴结单个核细胞诱导的C.B10及F1模型的发病时间一致[12]，在第12天时外周血象及股骨有核细胞计数均显著下降，符合骨髓衰竭小鼠模型的骨髓衰竭过程。同时实验中我们以流式细胞术在不同时间点追踪DsRed^+^细胞（PE荧光）在模型小鼠体内淋巴造血器官的分布过程，证实在DsRed小鼠淋巴细胞输注诱导的C.B10小鼠模型体内可以追踪供鼠来源的T淋巴细胞。同时，在骨髓衰竭晚期阶段我们分选了C.B10 AA小鼠骨髓、脾脏及淋巴结中供鼠来源的T淋巴细胞进行PCR芯片分析，结果显示骨髓中供鼠来源的 CD4^+^和CD8^+^ T细胞中CD38、IFN-γ、LAG3、CSF1、SPP1及TNFSF13B基因的mRNA表达增高，提示着细胞处于活化状态[13]–[14]。相反，骨髓中供鼠来源的CD4^+^ T细胞中FOXP3和CTLA4基因的mRNA表达降低，而这两个基因的表达减低则和细胞免疫抑制活性降低有关[15]–[16]。上述结果提示该动物模型中供鼠来源病理性T淋巴细胞具有更强的免疫活性，而免疫抑制活性则减弱。这与既往针对AA的相关研究的结果相一致[1],[8]，提示本模型符合AA的免疫学特点，可为AA动物模型中病理性T淋巴细胞的相关研究提供一个合理的实验工具。

T淋巴细胞可以分为naïve T和激活的T淋巴细胞，naïve T产生细胞因子的能力有限[17]，但接触抗原后naïve T可变成激活的T淋巴细胞，激活的T淋巴细胞包括T_EM_、T_CM_和T_EFF_，这些细胞是由它们不同的表型、功能和迁移模式定义的[18]。T_EFF_细胞群代表着最近激活的T淋巴细胞，并在初级免疫反应后被消除。而T_CM_则在二次免疫应答中能迅速增殖，并且其归巢和迁徙模式和naïve T一样。但T_CM_也表达炎性趋化因子受体，使其可以迁移到慢性炎症反应的部位[19]，这可能是我们实验结果中在骨髓衰竭晚期淋巴结中也可见到部分T_CM_的原因之一。

淋巴细胞归巢是指淋巴细胞经血液循环选择性穿越毛细血管高内皮微静脉后定向性迁移并定居于外周免疫器官或特定的组织区域的过程[20]。T淋巴细胞的归巢过程是受多种细胞黏附分子、趋化因子等参与并受各种因素调节的复杂过程[21]。细胞间黏附分子-1（ICAM-1/CD54）、淋巴细胞功能相关抗原-1（LFA-1）、整合素αLβ2、αMβ2和αxβ2等的表达均可影响T淋巴细胞的归巢能力[21]。在模型中我们研究了不同时间点DsRed^+^细胞在模型小鼠体内的分布，发现在骨髓衰竭的早期阶段，C.B10模型小鼠骨髓中的DsRed^+^ T细胞比例非常少，而脾脏和淋巴结中的比例相对较高，而在骨髓衰竭后期骨髓中的DsRed^+^ T细胞比例显著升高，提示该模型中供鼠来源的T淋巴细胞先归巢到外周淋巴器官，在其中扩增并分化，然后转运至骨髓。在整个骨髓衰竭的过程中，胸腺中DsRed^+^ T细胞均很少，说明胸腺对异体T淋巴细胞的归巢过程更具抵抗性，提示胸腺在免疫介导的AA小鼠模型的骨髓衰竭过程中可能是一个免疫豁免部位，这在既往研究中也被证实[22]。因为在AA小鼠形成骨髓衰竭的过程的早期阶段，在骨髓组织内仅发现少量DsRed细胞，不足以分析淋巴细胞的表型，而胸腺作为一个免疫豁免器官整个过程DsRed^+^ T淋巴细胞都很少，故在骨髓衰竭的晚期阶段即第9、12天我们分析了不同器官内的CD4^+^和CD8^+^ T细胞的CD44和CD62L的表达。我们的结果显示，在第9、12天时AA模型骨髓及脾脏中供鼠来源的CD4^+^或CD8^+^ T细胞主要是T_EM_，且骨髓中TEM的数量略高于脾脏中，而淋巴结中的供鼠来源的CD4^+^、CD8^+^ T细胞包括T_EFF_、T_EM_、T_CM_以及naïve T淋巴细胞，这一结果初步提示了骨髓中的供鼠来源淋巴细胞的免疫活性高于其他淋巴器官。研究提示记忆性T淋巴细胞会优先聚集于因抗原持续存在而导致慢性炎症的脾脏中，但如果感染或炎症过程是短暂的、急性的，则它们会优先定位于骨髓[23]–[24]。而本研究的小鼠模型正是根据组织相容性不合建立的动物模型，可以导致慢性炎症反应，使得T_EM_向骨髓中聚集。但同时也不能除外早期归巢到骨髓的少量DsRed^+^ T细胞自身在骨髓内由于异体抗原刺激下不断扩增引起细胞比例增加。然而，在我们的研究中并未观察到CD4^+^ T淋巴细胞和CD8^+^ T淋巴细胞两者之间的早期归巢过程的不同，但在骨髓衰竭的晚期阶段反而见到淋巴结中的CD8^+^ T细胞大部分为供鼠淋巴细胞，而CD4^+^ T细胞中供鼠淋巴细胞占不到一半的比例，这可能和异体淋巴细胞归巢和自体淋巴细胞归巢过程有差异有关，该现象的确切机制尚待进一步研究。

在本研究中我们还在建立的AA模型小鼠体内检测了骨髓、脾脏和淋巴结内特异性表达H60的CD8^+^细胞毒性T淋巴细胞（CD3^+^CD8^+^H60^+^）在T淋巴细胞中的比例，发现其在三个部位中的比例均随时间增加，且在骨髓衰竭的后期在AA模型的骨髓中的比例高于其他淋巴器官，和该模型的骨髓有核细胞计数及外周血象的下降程度相呼应，提示该类细胞在该模型的骨髓衰竭过程中起重要作用。

LAG3是一种类似CD4的检查点蛋白，通常在活化的效应T细胞（Teff）和调节性T细胞（Treg）上表达。LAG3的主要功能是调节免疫细胞，包括T细胞的活化、增殖、细胞因子的产生等。邵宗鸿团队的研究通过流式细胞术检测了重型AA患者和健康个体的淋巴细胞的LAG3表达水平，提示与健康对照组相比，重型AA患者CD4^+^ Teff、CD8^+^ Teff及Treg细胞中LAG3表达下降，且CD4^+^ Teff中LAG3的表达与CD4^+^/CD8^+^的比例正相关，与IL-2、IL-4、IFN-γ 和TNF-α的水平负相关。而经过免疫抑制治疗（IST）9个月后，与未治疗的重型AA及健康对照组相比，对IST有反应的重型AA患者CD4^+^ Teff、CD8^+^ Teff及Treg中LAG3表达上升。这些结果提示LAG3水平的下降可能导致CD4^+^ T细胞的扩增，Th1细胞的极化和CD8^+^T细胞的增殖[25]。而在本研究中，我们发现在骨髓衰竭晚期阶段，骨髓中供鼠来源的 CD4^+^和CD8^+^ T细胞中LAG3基因的mRNA表达增高。AA患者LAG3在蛋白和AA小鼠LAG3基因mRNA水平的变化不同，是由于其他原因导致了LAG3在蛋白水平的下降，例如：LAG3蛋白稳定性降低、泛素化导致的LAG3蛋白降解增快等，还是检测方法、检测时间点导致的结果不同还有待进一步的研究。

综上所述，DsRed转基因小鼠淋巴结细胞输注在C.B10小鼠体内可以诱导骨髓衰竭，成功建立可追踪供鼠来源的病理性T淋巴细胞的AA模型，有利于进一步阐明异常活化的T细胞在AA中的致病机制，但是在AA患者中病理性T细胞的特征是否与AA模型小鼠中一致还有待进一步研究。
